# Prevalence, associated factors, and short-term impact of central sensitization in high-altitude patients undergoing total knee arthroplasty

**DOI:** 10.1007/s00264-025-06560-x

**Published:** 2025-06-07

**Authors:** Siqing Zhang, Li Tan, Xuemei Bai, Linmei Deng, Sha Wan, Meng Ding, Kefu Lin, Zhen Tian, Lang Li

**Affiliations:** 1 Department of Neurosurgery, West China Hospital of Sichuan University, Chengdu, China; 2 Department of Orthopedics, Hospital of Chengdu Office of People’s Government of Tibetan Autonomous Region (Hospital.C.T.), Chengdu, China; 3 Department of Rehabilitation, Hospital of Chengdu Office of People’s Government of Tibetan Autonomous Region (Hospital.C.T.), Chengdu, China

**Keywords:** Central sensitization, High-altitude, Total knee arthroplasty, Knee osteoarthritis

## Abstract

**Purpose:**

Central sensitization (CS) plays a critical role in prolonged pain and poor outcomes after total knee arthroplasty (TKA), but its prevalence and impact in high-altitude populations remain unexplored. This study aims to examine the prevalence of CS, its associated factors, and short-term postoperative outcomes in high-altitude TKA patients.

**Methods:**

This prospective, single-centre cohort study included high-altitude (above 2,500 m) TKA patients with primary knee osteoarthritis (OA). Central sensitization (CS) was diagnosed using the Central Sensitization Inventory (CSI), with a cutoff score of ≥ 40. Propensity score matching (PSM) was applied to balance baseline characteristics between the CS and non-CS groups. Preoperative factors, postoperative outcomes (pain levels, complications, opioid consumption, hospital stay), and the incidence of chronic pain and dissatisfaction at six months were collected and analyzed using SPSS software.

**Results:**

A total of 230 patients were included, with 36 (15.7%) classified as having CS. Multivariable logistic regression identified female gender (OR: 3.9, 95% CI: 1.0–14.3, *P* = 0.043), higher body mass index (BMI) (OR: 1.2, 95% CI: 1.1–1.3, *P* = 0.006), and residence above 4,000 m (OR: 5.1, 95% CI: 1.7–15.1, *P* = 0.003) as significant factors associated with CS. After PSM, the CS group had significantly worse short-term outcomes, with higher pain scores at 24, 48, and 72 h (*P* < 0.001), increased incidence of postoperative nausea and vomiting (PONV) (*P* < 0.001), longer hospital stays (*P* < 0.001), greater cumulative opioid consumption (*P* < 0.001), higher rates of chronic postoperative pain (46.9% vs. 21.9%, *P* = 0.014), and greater patient dissatisfaction (25.0% vs. 6.3%, *P* = 0.039) compared to the non-CS group.

**Conclusion:**

This study found a 15.7% prevalence of CS among high-altitude TKA patients. Female gender, higher BMI, and residence at altitudes above 4,000 m were identified as factors associated with CS. Furthermore, CS was linked to worse short-term postoperative outcomes, including higher pain levels, increased incidence of PONV, greater opioid consumption, longer hospital stays, and a higher prevalence of chronic postoperative pain and dissatisfaction.

## Introduction

Pain management is crucial in total knee arthroplasty (TKA), a well-established treatment for end-stage knee osteoarthritis (OA). Despite advances in surgical techniques and pain management, about 20% of TKA patients experience prolonged postoperative pain and dissatisfaction [1]. Traditionally, OA pain was attributed to joint structural changes, with treatment focusing on nociceptive pain [2]. However, recent studies have found that structural damage is not directly correlated with pain severity [3, 4], and mechanisms such as central sensitization (CS) may play a more significant role in pain perception [5, 6].

CS involves heightened pain sensitivity due to altered processing in the central nervous system and can occur alongside nociceptive and neuropathic pain [7]. It results from mechanisms such as increased neuronal excitability and weakened inhibitory pathways [7]. Evidence indicates that CS affects 20–40% of OA patients, particularly those with knee OA [5, 6]. Preoperative CS is associated with longer hospital stays, chronic postoperative pain, worse functional outcomes, higher costs, and reduced surgical benefits in TKA patients [8–10]. These patients also tend to respond poorly to standard pain management strategies, like non-steroidal anti-inflammatory drugs (NSAIDs), and report higher dissatisfaction after surgery [11].

People living at high altitudes, including around 140 million residing above 2,500 m and over 12 million at elevations above 4,000 m, have a higher prevalence of symptomatic knee OA [12]. In these populations, knee disorders often develop earlier and persists longer, increasing the likelihood of undergoing TKA [13, 14]. Chronic exposure to hypoxic environments may lead to physiological adaptations, such as altered pain perception and shifts in pain thresholds, potentially influencing both peripheral and central pain mechanisms [15]. While some studies suggest that individuals newly exposed to high altitudes experience reduced pain thresholds due to acute hypoxia, long-term residents may develop higher pain thresholds [16]. However, these conclusions remain controversial. Despite the distinct characteristics of high-altitude populations, no studies have explored the prevalence or role of central sensitization (CS) in TKA patients living at high altitudes, highlighting a critical gap in current research.

This study aimed to examine the incidence of CS in this population, identify associated factors, and assess short-term postoperative outcomes following TKA.

## Methods

### Study design and ethics approval

This prospective, single-center cohort study was approved by our hospital’s Ethics Committee and conducted in accordance with the Declaration of Helsinki. Informed consent was obtained from all participants prior to data collection.

### Participants selection

Inclusion criteria: (1) age over 40 years; (2) unilateral TKA due to primary knee OA; (3) residence in high-altitude regions (above 2,500 m); (4) ability to understand and complete relevant questionnaires.

Exclusion criteria: (1) bilateral TKA; (2) concurrent serious medical conditions significantly affecting pain, such as cancer, spinal cord injury, or other neurological pain disorders; (3) history of psychiatric disorders; (4) history of drug or alcohol abuse; (5) preoperative use of medications for central sensitization, such as pregabalin or duloxetine; (6) opioid use within one month before surgery; (7) history of previous surgeries on the same knee; (8) American Society of Anesthesiologists (ASA) status classification of III or above.

### Confirmation of central sensitization

The Central Sensitization Inventory (CSI) was used to confirm the presence of central sensitization before surgery [17]. While quantitative sensory testing (QST) can assess central sensitization through external stimuli at affected or distant sites, it is costly, time-consuming, and invasive, limiting its practical application. The CSI, a validated tool for assessing musculoskeletal disorders, was chosen for this study due to its reliability, ease of use, and non-invasive nature, making it suitable for clinical practice [18]. It includes 25 items that evaluate the presence and severity of central sensitization symptoms using a 5-point Likert scale (0 = never, 1 = rarely, 2 = sometimes, 3 = often, 4 = always). A CSI score of ≥ 40 has been validated as the threshold for identifying central sensitization [18].

### Participants enrolment and grouping

**Fig. 1:**
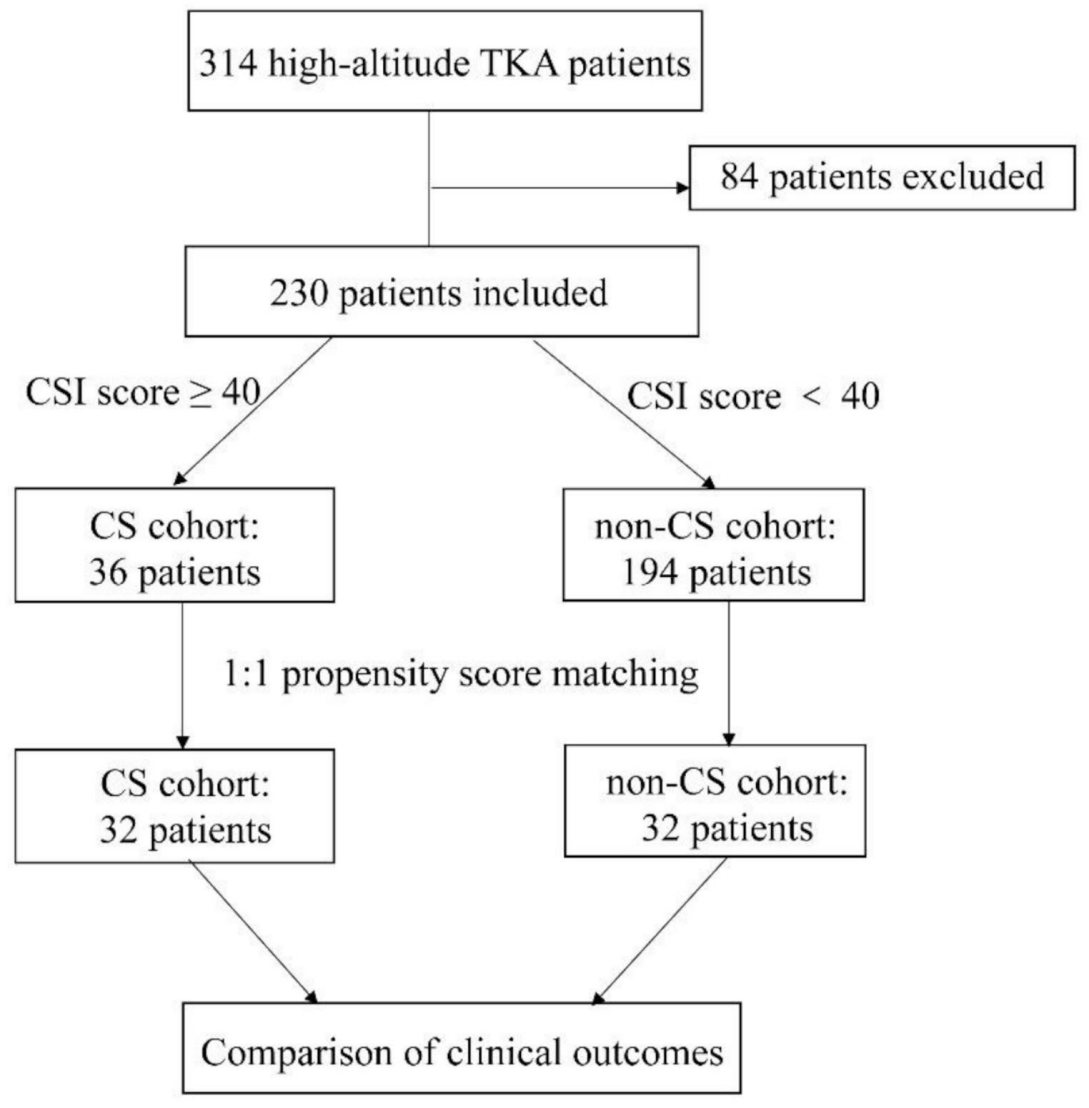
Participant enrolment and grouping flow diagram. TKA, total knee arthroplasty; CSI, Central Sensitization Inventory; CS, central sensitization

During the study period, 314 consecutive patients met the inclusion criteria. However, 84 patients were excluded based on the exclusion criteria. Specifically, 37 patients were unable or refused to complete the questionnaire, nine patients had neurological pain disorders, seven patients had a history of psychiatric disorders, five patients had a history of drug or alcohol abuse, ten patients had undergone previous surgical treatments on the affected knee, three patients had preoperative use of pregabalin, six patients had used opioids within one month prior to surgery, and seven patients had ASA status classification of III or above. Ultimately, 230 patients were included in the study, with 36 patients classified into the CS group (CSI score ≥ 40), while the remaining 194 patients were placed in the non-CS group (CSI score < 40). (Fig. 1).

To minimize potential confounding factors and ensure a balanced comparison between the CS and non-CS groups, propensity score matching (PSM) was applied. The propensity scores were calculated using a logistic regression model based on baseline covariates, including age, body mass index (BMI), gender, side of surgery, altitude of residence, occupation type, symptom duration, and preoperative VAS score. This matching process resulted in 32 patients in the CS group being matched with 32 patients in the non-CS group (Fig. 1).

### Data collection

Baseline data included the preoperative Central Sensitization Inventory (CSI) score, age, BMI, gender, side of surgery, altitude of residence, occupation type, symptom duration, Age-adjusted Charlson Comorbidity Index (ACCI), operative time, and preoperative pain level, assessed using the Visual Analog Scale (VAS, 0–10, 0 represents no pain and 10 represents the worst possible pain).

Postoperative data included VAS scores collected at 24, 48, and 72 h. The simplified Postoperative Nausea and Vomiting (PONV) impact scale [19], length of hospital stay, and cumulative opioid consumption were recorded at discharge, with opioid consumption calculated using a morphine milligram equivalent (MME) conversion tool. At the six month follow-up, chronic postoperative pain incidence and patient dissatisfaction were recorded. Chronic pain was defined as pain lasting at least three months after surgery, localized to the surgical area or related dermatomes, with other causes excluded [20].

### Surgical procedure and postoperative management

All procedures were performed by the same experienced surgeon under general anaesthesia, without the use of a tourniquet. Primary TKA was performed using a standard medial parapatellar approach, with a cemented posterior-stabilized implant.

A multimodal blood-loss prevention approach was employed, including preoperative optimization of haemoglobin levels and administration of tranexamic acid (TXA) at a dose of 15 mg/kg, 15 to 20 min prior to surgery. For anaesthesia, adductor canal nerve blocks (ACBs) were administered by the anesthesiologist under ultrasound guidance using a single injection of 0.2% ropivacaine (15–30 mL). A periarticular injection (PAI) of a 60 mL cocktail consisting of saline, morphine sulfate, betamethasone sodium phosphate, betamethasone acetate, and ropivacaine was given. Prior to implant placement, 20 mL of the cocktail was injected posterior to the joint capsule, and another 20 mL was used to infiltrate the medial and lateral collateral ligaments. After implant placement, the remaining 20 mL was injected into the quadriceps muscle, its associated ligamentous structures, and surrounding subcutaneous tissues.

Postoperatively, patients were encouraged to initiate knee mobilization on the first day after surgery and progressively increase participation in rehabilitation activities. Pain management followed a multimodal analgesia strategy to minimize opioid consumption. A patient-controlled analgesia (PCA) pump was used for personalized pain control during the first 24–48 h, with opioids reserved for breakthrough pain. Nonsteroidal anti-inflammatory drugs (NSAIDs) were prescribed regularly as part of the postoperative analgesia regimen.

For thromboembolic prophylaxis, low-molecular-weight heparin (LMWH) was administered for seven days, followed by direct oral anticoagulants (DOACs) for an additional two weeks. Routine opioid prescriptions were avoided, with opioids reserved for rescue analgesia based on individualized assessment. At discharge, patients were provided with oral NSAIDs for continued pain management.

### Statistical analysis

All statistical analyses were conducted using SPSS software, version 22.0 (SPSS Inc., Chicago, IL, USA). Results for continuous variables were expressed as mean ± standard deviation or median (interquartile range), depending on their distribution. Categorical variables were presented as frequencies and percentages. Continuous variables were analyzed using either t-tests or Mann-Whitney U tests, based on the outcomes of normality testing, while categorical variables were compared using chi-square (χ²) tests. PSM was employed to reduce the effects of confounding variables between the CS and non-CS groups. Logistic regression was used to estimate propensity scores based on selected covariates, and a 1:1 matching strategy with a nearest-neighbour approach and a caliper width of 0.2 was applied. Post-matching comparisons were performed to evaluate differences in baseline characteristics and postoperative outcomes between the two groups. Statistical significance was determined with a *P*-value threshold of < 0.05.

## Results

### Prevalence of central sensitization

A total of 230 patients were included in the study, with 36 patients classified into the CS group (CSI score ≥ 40), while the remaining 194 patients were placed in the non-CS group (CSI score < 40). Therefore, the prevalence of CS among these specific patients was 15.7% (36/230). Table 1 summarizes the baseline characteristics of all included patients.

**Table 1:** Baseline characteristics of patients

	Overall (*n* = 230)	CS cohort (*n* = 36)	Non-CS cohort (*n* = 194)	*P*
Preoperative CSI score, mean ± SD	20.2 ± 13.8	49.3 ± 5.1	14.7 ± 9.8	< 0.001
Age (years), mean ± SD	62.2 ± 8.5	61.2 ± 9.7	62.40 ± 8.3	0.446
BMI (kg/m^2^), mean ± SD	25.5 ± 4.2	26.4 ± 4.3	25.2 ± 4.1	0.004
Gender				0.008
Male	13 (20.3%)	5 (13.9%)	8 (36.6%)	
Female	51 (79.7%)	27 (86.1%)	24 (63.4%)	
Side				0.776
Right	120 (52.2%)	18 (50.0%)	102 (52.6%)	
Left	110 (47.8%)	18 (50.0%)	92 (47.4%)	
Altitude of residence				< 0.001
2,500-4,000 m	168 (73.0%)	16 (53.3%)	152 (78.4%)	
>4,000 m	62 (27.0%)	20 (46.7%)	42 (21.6%)	
Occupation				0.004
Physical laborers	135 (58.7%)	29 (80.6%)	106 (54.6%)	
Non- physical laborers	95 (41.3%)	7 (19.4%)	88 (45.4%)	
Symptom duration (years), median (IQR)	6.00 (4.0, 10.0)	10.00 (5.0, 10.0)	6.00 (3.0, 10.0)	0.026
ACCI, median (IQR)	2.0 (1.0, 3.0)	1.0 (0.0, 4.0)	2.0 (1.0, 3.0)	0.224
Operative time (minutes), mean ± SD	111.5 ± 34.1	113.7 ± 36.2	109.4 ± 32.8	0.583
Preoperative VAS, median (IQR)	4.0 (3.0, 5.0)	5.0 (4.0, 6.0)	3.0 (3.0, 4.0)	< 0.001

CS central sensitization, CSI Central Sensitization Inventory, BMI body mass index, ACCI Age-adjusted Charlson Comorbidity Index, VAS visual analog scale, SD standard deviation, IQR interquartile range

### Associated factors of central sensitization

In the multivariable logistic regression analysis, several factors were significantly associated with CS. A higher BMI, female gender, and residence at altitudes above 4000 m were identified as key predictors. Specifically, each 1 kg/m² increase in BMI raised the odds of CS by 1.2 times (OR: 1.2, 95% CI: 1.1–1.3, *P* = 0.006). Female patients were nearly four times more likely to have CS than males (OR: 3.9, 95% CI: 1.0–14.3, *P* = 0.043). Additionally, living above 4000 m increased the odds of developing CS by over five times compared to lower altitudes (OR: 5.1, 95% CI: 1.7–15.1, *P* = 0.003). Other factors such as age, surgical side, occupation, symptom duration, and preoperative VAS scores were not significantly associated with CS (Table 2).

**Table 2:** Multivariable logistic regression of factors associated with CS

	OR	95% CI	*P*
Age, ≥ 60 years vs. <60 years	1.9	0.5, 7.0	0.347
BMI, per 1 kg/m² increase	1.2	1.1, 1.3	0.006
Gender, female vs. male	3.9	1.0, 14.3	0.043
Side, left vs. right	1.5	0.5, 4.4	0.423
Altitude, > 4,000 m vs. 2,500-4,000 m	5.1	1.7, 15.1	0.003
Occupation, physical laborers vs. non-laborers	1.6	0.5, 5.5	0.454
Symptom duration, per 1-year increase	3.8	0.4, 38.9	0.795
Preoperative VAS score, per 1-point increase	2.5	0.3, 22.2	0.411

CS central sensitization, OR odds ratio, CI confidence Interval, BMI body mass index, VAS visual analog scale

### Clinical comparison of CS and non-CS cohorts

Following PSM, 64 patients were included, with 32 in the CS group and 32 in the non-CS group. The baseline characteristics between the two groups were comparable, with no significant differences in age, BMI, gender, side of surgery, altitude of residence, occupation, symptom duration, or preoperative VAS scores. This matching ensured a balanced comparison between groups for evaluating postoperative outcomes (Table 3).

**Table 3:** Baseline characteristics of patients after PSM

	Overall (*n* = 64)	CS cohort (*n* = 32)	Non-CS cohort (*n* = 32)	*P*
Age (years), mean ± SD	62.4 ± 8.8	62.0 ± 9.8	62.8 ± 7.8	0.714
BMI (kg/m^2^), mean ± SD	25.7 ± 4.4	26.1 ± 4.3	25.5 ± 4.3	0.407
Gender				0.351
Male	76 (20.3%)	5 (25.0%)	71 (15.6%)	
Female	154 (79.7%)	31 (75.0%)	123 (84.4%)	
Side				1.000
Right	34 (53.1%)	17 (53.1%)	17 (53.1%)	
Left	30 (46.9%)	15 (46.9%)	15 (46.9%)	
Altitude of residence				0.448
2,500-4,000 m	27 (42.2%)	15 (46.9%)	12 (37.5%)	
>4,000 m	37 (57.8%)	17 (53.1%)	20 (62.5%)	
Occupation				0.756
Physical laborers	51 (79.7%)	25 (78.1%)	26 (81.3%)	
Non- physical laborers	13 (20.3%)	7 (21.9%)	6 (18.7%)	
Symptom duration (years), median (IQR)	10.0 (4.8, 10.0)	10.0 (5.0, 10.0)	6.5 (4.0, 10.0)	0.297
Preoperative VAS, median (IQR)	5.0 (4.0, 6.0)	5.0 (4.0, 6.0)	5.0 (4.0, 5.3)	0.675

PSM propensity score matching, CS central sensitization, BMI body mass index, VAS visual analog scale, SD standard deviation, IQR, interquartile range

The comparison of postoperative outcomes showed that patients in the CS group had significantly worse short-term results. VAS scores were consistently higher at 24, 48, and 72 h postoperatively (*P* < 0.001). The CS group also had a higher incidence of PONV (median: 2.0 vs. 0.0, *P* < 0.001), a longer hospital stay (median: 9.0 vs. 7.0 days, *P* < 0.001), and greater cumulative opioid consumption during hospitalization (median MME: 100.0 vs. 30.0 mg, *P* < 0.001). Additionally, chronic postoperative pain was more prevalent in the CS group (46.9% vs. 21.9%, *P* = 0.014), and patient dissatisfaction was higher (25.0% vs. 6.3%, *P* = 0.039) (Table 4).

**Table 4:** Comparison of postoperative clinical outcomes between CS and non-CS groups after PSM

	CS cohort (*n* = 32)	Non-CS cohort (*n* = 32)	*P*
VAS at postoperative 24 h, median (IQR)	4.0 (3.0, 4.0)	2.0 (2.0, 3.0)	< 0.001
VAS at postoperative 48 h, median (IQR)	3.0 (3.0, 3.0)	2.0 (2.0, 2.0)	< 0.001
VAS at postoperative 72 h, median (IQR)	3.0 (2.0, 3.0)	2.0 (1.0, 2.0)	< 0.001
Simplified PONV impact scale, median (IQR)	2.0 (1.0, 2.3)	0.0 (0.0, 1.0)	< 0.001
Length of hospital stay (days), median (IQR)	9.0 (9.0, 11.0)	7.0 (7.0, 8.0)	< 0.001
MME consumption during hospital stay (mg), median (IQR)	100.0 (60.0, 130.0)	30 (0.0, 50.0)	< 0.001
Chronic postoperative pain	15 (46.9%)	7 (21.9%)	0.014
Dissatisfaction	8 (25.0%)	2 (6.3%)	0.039

CS central sensitization, PSM, propensity score matching, VAS visual analog scale, PONV postoperative nausea and vomiting, MME morphine milligram equivalent, SD standard deviation, IQR interquartile range

## Discussion

This study is the first to examine the prevalence of CS, its associated factors, and its impact on clinical outcomes in high-altitude TKA patients. We found that 15.7% of these specific patients exhibited CS, as measured by the CSI. Patients with CS were more likely to be female, have higher BMIs, and reside at altitudes above 4,000 m. In terms of short-term outcomes, the CS group experienced significantly worse postoperative results, including higher pain levels at 24, 48, and 72 h, more PONV, increased opioid consumption, longer hospital stays, and a higher incidence of chronic postoperative pain and dissatisfaction.

This study is the first to investigate the relationship between altitude and CS in TKA patient. We observed an overall CS prevalence of 15.7%, which is lower than the 20–40% typically reported in non-altitude-specific populations [5, 6]. However, when stratifying the population by altitude, notable differences emerged. Among patients living at altitudes between 2,500 and 4,000 m, the prevalence of CS was 9.5% (16/168). In contrast, for those residing above 4,000 m, the prevalence significantly increased to 32.3% (20/62). Our multivariable logistic regression analysis identified altitude as a significant factor in CS development. Patients living at elevations above 4,000 m were more than five times more likely to develop CS compared to those at lower altitudes (OR: 5.1, 95% CI: 1.7–15.1, *P* = 0.003), indicating a strong relationship between extreme altitude and the risk of CS.

Previous research provides some insight into this phenomenon. Individuals from low-altitude regions tend to experience a substantial reduction in pain thresholds, up to 30%, when they first ascend to high altitudes due to acute hypoxia, which can increase pain sensitivity [21]. In contrast, long-term residents of moderate high altitudes, such as between 2,500 and 4,000 m, typically develop higher pain thresholds [16], offering some degree of protection against the development of CS. This corresponds to the relatively lower prevalence of CS at these altitudes in our study. However, at extreme altitudes above 4,000 m, this protective effect appears to diminish. Long-term exposure to severe hypoxia at such elevations may alter pain processing in the central nervous system, aggravate joint inflammation, and accelerate the progression of osteoarthritis [22]. In addition, residents of remote, high-altitude regions may have limited access to medical care, leading to inadequate treatment of pain, which could further contribute to the heightened risk of developing CS.

In addition to altitude, we found that female gender and higher BMI are associated with central sensitization. Females, who are generally more prone to chronic pain conditions and often exhibit heightened pain sensitivity, have been shown to have a higher prevalence of CS [23]. Similarly, elevated BMI not only increases mechanical stress on the joints but is also linked to metabolic disorders, both of which contribute to the progression of osteoarthritis and are well-established risk factors for CS [24]. These associations are consistent with existing literature on CS risk factors, reinforcing the alignment of our findings with previous studies.

Our postoperative findings align with previous research, emphasizing the negative impact of CS on surgical outcomes [8–11]. In this study, patients with CS reported significantly higher pain levels at 24, 48, and 72 h post-surgery, along with more episodes of PONV, longer hospital stays, and greater opioid consumption. Additionally, the higher incidence of chronic postoperative pain and dissatisfaction in the CS group supports the idea that CS prolongs pain sensitization and recovery. Studies have shown that knee OA patients with preoperative CS experience worse pain scores, lower patient-reported outcomes, and higher dissatisfaction rates two years after TKA. Moreover, CS has been linked to an increased risk of wound complications and persistent pain following both primary and revision TKA [8–11]. These findings underscore the importance of preoperative CS assessments for identifying at-risk patients, managing expectations, and optimizing postoperative recovery to reduce complications.

While our study provides valuable insights into CS in high-altitude TKA patients, several limitations should be noted. First, the study was conducted at a single centre with a relatively small sample size, which may limit the generalizability of our findings to other high-altitude populations, especially considering potential institutional biases. Second, although the CSI is a validated tool for assessing CS, it remains inherently subjective. More objective methods, such as QST, could provide a more comprehensive evaluation of CS severity, but resource limitations prevented their use in this study. Additionally, our follow-up period focused primarily on short-term postoperative outcomes, which may not fully capture the long-term impact of CS on recovery or the incidence of complications over time. Furthermore, due to the constraints of our prospective cohort design, we did not collect standardized intraoperative data, such as blood loss, despite evidence suggesting that high-altitude populations have an increased risk of intraoperative bleeding, which may influence postoperative outcomes [25]. Finally, the study design did not investigate potential interventions for CS, leaving the effects of treatment options unexamined.

## Conclusion

This study found a 15.7% prevalence of CS among high-altitude TKA patients. Female gender, higher BMI, and residence at altitudes above 4,000 m were identified as factors associated with CS. Furthermore, CS was linked to worse short-term postoperative outcomes, including higher pain levels, increased incidence of PONV, greater opioid consumption, longer hospital stays, and a higher prevalence of chronic postoperative pain and dissatisfaction.

## Data Availability

The research data supporting the results of this manuscript are available upon reasonable request from the corresponding author.
